# Exploring the Interrelationship Between Diabetes Mellitus and Chronic Lung Diseases: A Comprehensive Review

**DOI:** 10.7759/cureus.69617

**Published:** 2024-09-17

**Authors:** Pakeeza Tarar, Venkata Varshitha Bandi, Hooria Sarwar, Rafik Maged, Mohit Sinha, Hema Manvi Koneru, Iana Malasevskaia

**Affiliations:** 1 Internal Medicine, California Institute of Behavioral Neurosciences & Psychology, Fairfield, USA; 2 Medical Research, California Institute of Behavioral Neurosciences & Psychology, Fairfield, USA; 3 Psychiatry, California Institute of Behavioral Neurosciences & Psychology, Fairfield, USA; 4 Obstetrics and Gynecology, Private Clinic 'Yana Alexandr', Sana'a, YEM; 5 Research and Development, California Institute of Behavioral Neurosciences & Psychology, Fairfield, USA

**Keywords:** systematic review, copd, chronic obstructive pulmonary disease, asthma, chronic lung diseases, t2dm, type 2 diabetes, t1dm, type 1 diabetes, diabetes mellitus

## Abstract

Diabetes mellitus (DM) is a chronic metabolic disorder that affects millions of individuals worldwide. With an increasing prevalence, understanding its implications for respiratory health is essential. Chronic lung diseases, such as asthma and chronic obstructive pulmonary disease (COPD), significantly impact morbidity and healthcare costs, with COPD alone accounting for substantial economic burdens. This comprehensive review investigates the intricate relationship between DM and chronic lung diseases. A systematic search across multiple databases yielded 1,078 articles, from which 10 studies were selected for detailed examination. The findings reveal a bidirectional relationship: diabetes increases the risk of developing chronic lung conditions, while chronic lung diseases can exacerbate glycemic control. Shared inflammatory pathways and comorbidities complicate patient outcomes, underscoring the urgent need for integrated treatment approaches. By elucidating the mechanisms linking these conditions, this review provides valuable insights for healthcare professionals, emphasizing the importance of interdisciplinary care to enhance the quality of life for individuals affected by both diabetes and chronic lung diseases. The results highlight the necessity for further research to explore targeted therapies and preventive measures addressing these interconnected health issues.

## Introduction and background

Diabetes mellitus (DM) is defined as a chronic metabolic disorder that is characterized by the body's incapacity to produce or effectively utilize insulin, a hormone that is crucial for controlling blood glucose levels [1]. The prevalence of diabetes has surged in recent years, with the estimation that 537 million individuals aged 20-79 are living with the condition in 2021, equating to one in 10 individuals [1]. Projections indicate that this number could jump to 643 million by the year 2030 and 783 million by 2045. The economic impact of diabetes is staggering, with health expenditures reaching at least $966 billion globally, reflecting a 316% increase over the past 15 years [1].

Chronic lung diseases, including asthma, chronic obstructive pulmonary disease (COPD), and idiopathic pulmonary fibrosis (IPF), are often the result of inflammatory changes in the lungs that lead to pulmonary dysfunction. COPD, one of the most prevalent chronic lung diseases, affects an estimated 10.3% of the population. The economic burden associated with COPD is significant; in the European Union, 6% of the total healthcare budget is allocated to respiratory disease management, with COPD accounting for 56% of these costs [2]. In the United States, projected costs for COPD management are expected to escalate to $40 billion annually over the next 20 years [2]. Asthma, another chronic lung condition, impacts over 339 million individuals worldwide, with its prevalence steadily increasing over the past three decades [3].

DM is widely acknowledged for its association with various complications, including cardiovascular diseases, renal dysfunction, peripheral neuropathy, and vision impairment [4]. Recently, the intersection between diabetes and chronic lung diseases has garnered significant attention, particularly as the prevalence of respiratory conditions like asthma and COPD continues to rise, adding an economic burden to health care. In 2020, the 20-year direct medical costs imputed to COPD were estimated to be $800.90 billion [5]. A population-based retrospective study in Italy found that the prevalence of type 2 DM (T2DM) among COPD patients was notably higher (18.7%) compared to the general population (10.5%) [6]. Additionally, another study revealed an increased risk of asthma in individuals with T2DM, with prevalence rates of 13.5% in men and 16.6% in women, compared to 7.5% and 9.6% in their non-diabetic counterparts, respectively [7].

Despite significant advances in understanding the individual pathophysiology of diabetes and chronic lung diseases, their interconnectedness remains inadequately explored. Systemic inflammation and oxidative stress, commonly associated with diabetes, are known to exacerbate pulmonary conditions while chronic lung diseases can worsen glycemic control; however, comprehensive studies addressing this relationship are limited [4]. This gap in the literature underscores the need for further research to elucidate the underlying mechanisms linking these comorbidities.

This review aims to investigate the relationship between DM and the progression of chronic lung diseases, including asthma and COPD. By thoroughly analyzing clinical data and patient outcomes, we seek to enhance our understanding of how diabetes influences respiratory function and how chronic lung diseases correlate with poor glycemic control. Understanding the nexus between these two prevalent health issues is crucial for developing integrated treatment approaches.

By addressing this critical gap, this research contributes to the broader field of chronic disease management, emphasizing the necessity for interdisciplinary approaches to patient care that ultimately enhance the quality of life for individuals suffering from both diabetes and chronic lung conditions.

## Review

This literature review aimed to investigate the association between DM (type 1 and type 2) and the risk of developing chronic lung diseases. The review was structured according to the Population, Intervention, Comparison, Outcomes and Study (PICOS) framework, focusing on a population of individuals with T1DM and T2DM and a comparator group of non-diabetic individuals. The outcomes of interest included the relationship between diabetes and the incidence and prevalence of chronic lung diseases such as COPD and asthma.

A systematic search was conducted across multiple electronic databases, including PubMed, Cochrane Library, and Europe PubMed Central (PMC), using a comprehensive set of keywords and Medical Subject Headings (MeSH) terms related to diabetes and chronic lung diseases. The search strategy was designed to capture observational studies, specifically cohort, case-control, and cross-sectional studies, and was published from inception in English (Table 1).

**Table 1:** Search strategy Medical Subject Headings (MeSH)

Search Strategy	Databases/Registers	Results Before filters/After filters	Filters
("Diabetes mellitus type 1"[All Fields] OR "T1DM"[All Fields] OR "Insulin dependent diabetes mellitus"[All Fields] OR "young onset diabetes mellitus"[All Fields] OR "DM Type 1"[All Fields] OR "Insulin dependent diabetes mellitus"[All Fields] OR "diabetes mellitus, type 1"[MeSH Terms] OR "diabetes mellitus, type 1/complications"[MeSH Terms] OR ("diabetes mellitus type 2"[All Fields] OR "T2DM"[All Fields] OR “non insulin dependent diabetes mellitus"[All Fields] OR "non insulin dependent diabetes mellitus"[All Fields] OR "DM type 2"[All Fields] OR "diabetes mellitus, type 2"[MeSH Terms] OR "diabetes mellitus, type 2/complications"[MeSH Terms])) AND ((("chronic"[All Fields] OR "chronical"[All Fields] OR "chronically"[All Fields] OR "chronicities"[All Fields] OR "chronicity"[All Fields] OR "chronicization"[All Fields] OR "chronics"[All Fields]) AND ("lung diseases"[MeSH Terms] OR ("lung"[All Fields] AND "diseases"[All Fields]) OR "lung diseases"[All Fields] OR ("lung"[All Fields] AND "disease"[All Fields]) OR "lung disease"[All Fields])) OR "asthma"[All Fields] OR "COPD"[All Fields] OR "obstructive pulmonary disease"[All Fields] OR "Emphysema"[All Fields] OR "Chronic Bronchitis"[All Fields] OR "Lung Fibrosis"[All Fields] OR "Cystic fibrosis"[All Fields] OR "pulmonary disease, chronic obstructive"[MeSH Terms] OR "asthma"[MeSH Terms] OR "Pulmonary fibrosis"[MeSH Terms])	PubMed/Medline	2660/184	Full text, Clinical Study, Observational Study, Humans, English.
(( “Diabetes mellitus type 1” OR “T1DM” OR “Insulin dependent diabetes mellitus” OR “Young onset diabetes mellitus” OR “DM type 1” OR “Insulin dependent diabetes mellitus”) OR (“Diabetes mellitus type 2” OR “T2DM” OR “Non insulin dependent diabetes mellitus” OR “Non insulin dependent diabetes mellitus” OR “ DM type 2”)) AND (‘’Chronic lung disease’’ OR “Asthma” OR “COPD” OR “ Obstructive pulmonary disease” OR “Emphysema” OR “ Chronic bronchitis” OR” Lung fibrosis” OR “Cystic fibrosis”) AND (Cohort OR ''Case Control'' OR ''Cross Sectional'') NOT ( ''Review'' OR ''meta-analysis'')	Europe PMC	556	No filters applied as all are research articles
Date Run: 05/07/2024 22:07:49 Comment: ID Search Hits #1 (“Diabetes mellitus type 1” OR “T1DM” OR “Insulin dependent diabetes mellitus” OR “Young onset diabetes mellitus OR “DM type 1” OR “Insulin dependent diabetes mellitus”):ti,ab,kw (Word variations have been searched) 33184 #2 MeSH descriptor: [Diabetes Mellitus, Type 1] explode all trees 7612 #3 #1 OR #2 33185 #4 MeSH descriptor: [Diabetes Mellitus, Type 2] explode all trees 26412 #5 ("diabetes mellitus type 2" OR T2DM):ti,ab,kw 33118 #6 #4 OR #5 33119 #7 (''Chronic obstructive pulmonary disease'' OR ''COPD'' OR Asthma OR Emphysema OR ''Bronchitis, Chronic'' OR ''Lung fibrosis'' OR ''Cystic fibrosis''):ti,ab,kw (Word variations have been searched) 68890 #8 MeSH descriptor: [Pulmonary Disease, Chronic Obstructive] explode all trees 8294 #9 #7 OR #8 68890 #10 #3 OR #6 53236 #11 #10 AND #9 275	Cochrane Library (CENTRAL)	275/263	Trials, Language : English
(( “Diabetes mellitus type 1” OR “T1DM” OR “Insulin dependent diabetes mellitus” OR “Young onset diabetes mellitus” OR “DM type 1” OR “Insulin dependent diabetes mellitus”) OR (“Diabetes mellitus type 2” OR “T2DM” OR “Non insulin dependent diabetes mellitus” OR “ Non insulin dependent diabetes mellitus” OR “ DM type 2”)) AND (‘’Chronic lung disease’’ OR “Asthma” OR “COPD” OR “ Obstructive Pulmonary disease” OR “Emphysema” OR “ Chronic Bronchitis” OR” Lung fibrosis” OR “Cystic fibrosis”)	EBSCO Open Dissertations	11	No filters applied
((“Diabetes mellitus type 1” OR “Insulin dependent diabetes mellitus ” OR “Young onset diabetes mellitus”) OR (“Diabetes mellitus type 2” OR “T2DM” OR “ DM Type2”)) AND (‘’Chronic lung disease’’ ) AND (''case control'' OR Cohort )	Science Direct	9246/52	Research articles Medicine and Dentistry English Open access & Open archive Search strategy in Title/abstract only: Not all fields.
Condition/disease: ( Diabetes mellitus) AND (chronic obstructive pulmonary disease)	ClinicalTrilas.gov Register	102/7	| Completed studies | Interventional, Observational studies | Studies with results

Inclusion criteria were established to ensure the relevance of studies: participants must have T1DM or T2DM, studies must be observational in design, and outcomes must be limited to specific chronic lung diseases. Studies that did not meet these criteria, including randomized controlled trials, ongoing studies, and non-human studies, were excluded.

The Rayyan app® (Rayyan, Cambridge, MA, USA) [8] was employed to manage and screen the identified articles efficiently. After removing duplicates, a total of 1,078 articles were screened. Following the application of inclusion and exclusion criteria, 52 articles were included for further analysis, of which 38 had full texts available for review. Ultimately, 10 of the most relevant articles were selected for detailed examination (Table 2).

**Table 2:** Summary of included studies COPD: Chronic Obstructive Pulmonary Diseases;  BMI: Body Mass Index; T2DM: Type 2 Diabetes Mellitus; AECOPD: Acute Exacerbation of Chronic Obstructive Pulmonary Disease; BMI: Body Mass Index; SES: Socio-economic status; SBP: Systolic blood Pressure; PXS: Polyexposure risk scores; PGS: Polygenic risk scores; HR: Hazard Ratio; T1D: Type 1 Diabetes; ICS: Inhaled corticosteroids; ASCVD: Atherosclerotic Cardiovascular Disease; RR: Relative Risk; T1DM: Type 1 Diabetes Mellitus; CI: Confidence Interval; HbA1c: Glycated Hemoglobin; OR: Odds ratio.

Author/Year	Type of Study	Sample size	Aim of study	Key findings
Song et al., 2010 [9]	Prospective Cohort study	38,570	To investigate the association between chronic airway inflammation (asthma or COPD) and T2DM in women.	38,570 women (aged ≥ 45 years) were divided on the basis of the presence or absence of Asthma or COPD and were followed for 12.2 years; T2DM was documented in 2,472 women. Those with asthma had a RR of 1.37 (95% CI, 1.20-1.57), as compared to RR of 1.38 (95% CI, 1.14-1.67) in women with COPD. No effect of age, smoking status, or BMI on associations was reported.
Stallberg et al., 2020 [10]	Cross-sectional study	7808	To assess the association between ICS use and the risk of T2DM in Swedish patients with COPD using real-world data.	T2DM prevalence was 5.9%. The 5-year cumulative incidence rate of T2DM was 1506.9 per 100,000 person-years High-dose ICS use was associated with a 64% increased risk of T2DM compared to non-ICS users, while low-dose ICS showed a 32% increase. The study confirms that ICS treatment, especially at high doses, may elevate the risk of developing T2DM in COPD patients.
Lin et al., 2021 [11]	Prospective observational study	196	To estimate the influence of both previously and newly diagnosed T2DM on outcomes in patients with AECOPD.	T2DM prevalence was 26%. Newly diagnosed T2DM patients had significantly lower (PaO2 compared to non-diabetics. T2DM patients experienced longer hospital stays. There were certain risk factors, like age, required assisted ventilation, higher troponin levels, and elevated fasting blood sugar levels, which increased mortality in patients hospitalized with COPD exacerbation.
Gonçalves et al., 2020 [12]	Prospective observational study	121	To evaluate the burden of hospital admission due to COPD exacerbations and concomitant T2DM.	121 COPD patients, with 47 (38%) having concomitant T2DM were analyzed, and the significant increase in hospital admissions due to AECOPD in patients with diabetes was reported (OR 2.66; P = 0.031), though no significant effect on the total COPD exacerbations was reported.
Papathanassiou et al., 2021 [13]	Prospective observational study	156	To inspect the prognostic value of HbA1c and adjusted glycemic variables in patients with AECOPD and their impact on morbidity and mortality over one year.	Out of 74.4% of men, 21.8% had T2DM. Median HbA1c was 5.9 (IQR: 5.4, 6.5) Lower HbA1c values were associated with a higher need for mechanical ventilation (median: 5.3 vs. 5.9, p = .038) No significant differences were found in hospitalization duration, mortality, or exacerbation events over the following year. HbA1c-adjusted glycemic variables showed no statistical significance.
Hsiao et al., 2015 [14]	Retrospective cohort study	17725	To examine the associations between T1DM and asthma.	The T1DM cohort reported 47% higher asthma incidence than in the control cohort (6.49 vs 4.42 per 1000 person-y), with an adjusted HR of 1.34 (95% [CI] = 1.11–1.62) while the HR increased to 17.4 (95% CI = 12.9-23.6) for T1DM patients who had more than two emergency department visits.
He and Patel 2022 [15]	Cross-sectional Study	21,935	To investigate whether the whether diabetes shares risk factors with other chronic conditions.	The data analyzed T2DM and seven associated chronic diseases. Among other risk factors, PXS risk and PGS risk scores were reported to have a higher impact and association with all seven diseases, including COPD (HR of 2.82) The study concludes that T2D shares PXS risks with other chronic diseases.
Luijks et al., 2015 [16]	Prospective Cohort study	610	To inquire about the association between COPD, glycemic control (HbA1C), and systolic blood pressure in diabetic patients.	The prevalence of COPD was reported to be 10.3% in diabetic patients. However, the association between COPD and HbA1C was not reported to be significant. [(p=0.54) or SBP (p=0.33)]
Ghafil et al., 2022 [17]	Case Control Study	200	To find out the prevalence of comorbidities among individuals with COPD and the association between these comorbidities and the severity of the disease.	The higher prevalence of T2DM, ASCVD, hypertension, and dyslipidemia was reported in COPD patients and high disease severity was also reported for COPD patients with these comorbidities.
Hörtenhuber et al., 2017 [18]	Prospective cohort study	51,926	To investigate the prevalence of asthma in young patients with T1DM and its impact on metabolic control.	T1D patients (<20 years);(3.4%) had asthma. Asthma patients required higher doses of insulin.They were more often male (61% vs 52%, P < .01) and experienced more severe hypoglycemia (4.5 vs 3.2 events/100 pts. years, P < .01). No overall difference in HbA1c was found, with minor influences on metabolic control and complications.

Discussion

This comprehensive review synthesizes findings from 10 relevant studies examining the association between DM and chronic lung diseases. The evidence indicates a bidirectional relationship where the presence of diabetes increases the risk of developing chronic lung conditions, and vice versa. This discussion explores the implications of these findings, the underlying mechanisms, and the potential for future research.

Diabetes as a Risk Factor for Chronic Lung Diseases

Several studies highlight the increased risk of T2D among patients with chronic lung diseases. For instance, Song et al. reported that women with COPD had a relative risk (RR) of 1.38 for developing T2D, while those with asthma had a RR of 1.37 [9]. This suggests that chronic airway inflammation may contribute to metabolic dysregulation, potentially through shared inflammatory pathways. The chronic inflammatory state seen in COPD may lead to insulin resistance, a hallmark of T2D, thereby exacerbating both conditions.

Stallberg et al. documented a 5.9% prevalence of T2D among COPD patients, with a significant cumulative incidence rate of 1506.9 per 100,000 person-years [10]. This finding underscores the importance of monitoring glycemic control in patients with COPD, as the inflammatory processes in chronic lung disease may exacerbate metabolic conditions. The overlap in risk factors, such as obesity and physical inactivity, further complicates this relationship.

Comorbidities and Their Impact on Patient Outcomes

The presence of comorbidities significantly impacts the health outcomes of patients with both COPD and diabetes. Lin et al. found that patients with acute exacerbations of COPD (AECOPD) and T2D experienced longer hospital stays and lower arterial oxygen levels (PaO2). This suggests that T2D may complicate the clinical course of COPD, leading to poorer respiratory function and increased healthcare utilization [11].

Similarly, Gonçalves et al. established that T2D increased the risk of hospital admissions due to COPD exacerbations (OR 2.66; P = 0.031), indicating a complex interplay between these conditions [12]. The increased risk of hospitalization highlights the need for integrated care approaches that address both diabetes management and respiratory health.

Conversely, not all studies found a direct association between diabetes and poor outcomes in COPD patients. For example, Papathanassiou et al. reported that HbA1c levels on admission did not predict exacerbation rates or mortality in AECOPD patients [13]. This highlights the need for further research to clarify the role of glycemic control in managing COPD and whether other factors, such as acute inflammatory responses, play a more significant role in patient outcomes.

Role of Inflammation

The inflammatory nature of both diabetes and chronic lung diseases suggests a shared pathophysiological mechanism. Chronic low-grade inflammation is a characteristic of both conditions, which may lead to a vicious cycle of worsening metabolic control and lung function. Inflammatory cytokines associated with T2D, such as tumor necrosis factor-alpha (TNF-α) and interleukin-6 (IL-6), may exacerbate airway inflammation in COPD, contributing to the progression of both diseases [4].

This is supported by the findings from Hsiao et al. [14], which pointed out that patients with diabetes had a higher incidence of asthma as compared to non-diabetics. The study reported a 47% higher incidence of asthma in T1DM patients compared to controls, suggesting that diabetes-related inflammation may predispose individuals to respiratory conditions. Understanding these inflammatory pathways could lead to targeted therapies that address both diabetes and chronic lung diseases.

Genetic and Environmental Factors

Emerging evidence suggests that genetic and environmental factors may contribute to the shared risks of T2D and chronic lung diseases. He and Patel utilized polygenic risk scores (PGS) and polyexposure risk scores (PXS) to demonstrate that individuals in the highest deciles of these scores had significantly increased risks of developing chronic diseases, including COPD and obesity [15]. This finding points to the potential for shared genetic predispositions that warrant further investigation.

Additionally, environmental factors such as smoking, air pollution, and socioeconomic status may play critical roles in the development of both T2D and chronic lung diseases. The interaction between these factors and genetic predispositions could provide a more comprehensive understanding of the pathways leading to these comorbidities.

Implications for Clinical Practice

The bidirectional relationship between diabetes and chronic lung diseases has important implications for clinical practice. Healthcare providers should adopt a holistic approach to patient management, recognizing the interconnectedness of these conditions. Regular screening for diabetes in patients with COPD and vice versa could lead to earlier interventions and improved outcomes.

Moreover, lifestyle modifications aimed at reducing inflammation-such as smoking cessation, weight management, and physical activity be emphasized in treatment plans for patients with either condition. Educational programs that inform patients about the risks of comorbidities could empower them to take an active role in managing their health.

Comparison With Other Evidence

Our review highlights a significant association between DM and chronic lung diseases, particularly COPD and asthma. This aligns with findings from several systematic reviews and meta-analyses, reinforcing the evidence that diabetes exacerbates the course of chronic lung conditions. For instance, a meta-analysis by Peng et al. analyzed 13 cohort studies involving 307,335 incident T2DM cases, reporting a RR of 1.25 (95% CI 1.16-1.34) for developing T2DM in patients with COPD compared to those without. Similarly, our findings indicated a bidirectional relationship, where chronic lung diseases significantly increase the risk of developing diabetes [19]. This convergence of results underscores the importance of monitoring glycemic control in patients with COPD.

While our review synthesized findings from diverse populations, some systematic reviews, such as those by Peng et al. [19] and Bai et al. [20], primarily focused on specific cohorts (e.g., COPD or IPF patients). This narrower focus may limit the generalizability of their findings across different chronic lung conditions. In contrast, our review encompasses a broader spectrum of chronic lung diseases, providing a more comprehensive understanding of the interplay between diabetes and respiratory health. Furthermore, our review utilized a variety of observational studies, including prospective cohort studies and case-control studies, to establish associations between diabetes and chronic lung diseases. In contrast, some systematic reviews relied heavily on case-control designs, which may introduce bias and limit causal inference. This methodological variation could lead to differences in the strength of the associations reported.

Furthermore, our comprehensive review corroborates and expands upon other previous reviews, including those by Torres et al. [21], Park et al. [22], and Gläser et al. [23], highlighting the bidirectional relationship and intertwined mechanisms wherein these chronic conditions influence and exacerbate one another.

Torres et al. elaborated on the association between asthma and T2DM, attributing it to systemic low-grade inflammation and the effects of corticosteroid therapy [21]. Our findings reinforce this connection, suggesting that the systemic inflammatory milieu prevalent in diabetes significantly undermines pulmonary function. Indeed, chronic inflammation, characterized by elevated pro-inflammatory cytokines such as TNF-α and IL-6, is not only a hallmark of diabetes but also contributes to pulmonary dysfunction, creating a vicious cycle that impairs both respiratory and metabolic health. This dual burden leads to a complex clinical scenario where managing asthma in the presence of diabetes becomes increasingly challenging, echoing Torres et al.'s assertion that the burden of dual diseases can severely impact patient outcomes [21].

In relation to COPD, Park et al. underscored the higher prevalence of diabetes among COPD patients and identified shared risk factors, notably obesity and smoking, that complicate management strategies [22]. Our review builds on this premise, confirming that diabetes not only increases the susceptibility to COPD but that the inverse holds as well; COPD may lead to deteriorating glycemic control. Similar findings were reported by Song et al., which indicated that women with COPD had a RR of 1.38 for developing T2DM compared to non-COPD patients [9]. The impact of these comorbidities is further manifested in Lin et al., who found that patients with newly diagnosed T2DM exhibited significantly lower oxygen levels and longer hospital stays during acute exacerbations of COPD [11]. Both conditions share a plethora of risk factors and physiological pathways that warrant further exploration. The challenge remains in elucidating direct mechanistic links, a gap also noted by Park et al. [22]. Our observations suggest that a comprehensive understanding of their bidirectional relationship is critical for developing effective treatment protocols.

Furthermore, Gläser et al. articulated how diabetes could exacerbate COPD progression, potentially through hyperglycemia's negative impacts on lung physiology and the inflammatory process [23]. Our findings concur with this perspective, emphasizing that chronic activation of inflammatory pathways due to diabetes can significantly worsen the clinical course of COPD. Supporting this, Gonçalves et al. reported a significant increase in hospital admissions due to acute exacerbations of COPD (AECOPD) among patients with coexisting T2DM, with an odds ratio of 2.66 [12]. This indicates that the interplay between these conditions indeed leads to a higher burden of hospitalizations and complications, echoing our conclusion about the need for integrated management strategies.

Importantly, these studies collectively illuminate a crucial gap in current clinical practices: the need for integrative treatment approaches. For instance, Stallberg et al. demonstrated that high-dose inhaled corticosteroids (ICS), commonly used in COPD management, were associated with a 64% increased risk of T2DM, bringing attention to potential medication-induced complications that necessitate careful consideration in patient management [10]. Our review advocates for coordinated care that bridges the disciplines of endocrinology and pulmonology, as interdisciplinary programs may play a pivotal role in lessening the burden of multiple chronic conditions. As supported by the insights from Torres et al. [21], Park et al. [22], and Lin et al. [11], the implementation of comprehensive management strategies tailored to the unique needs of patients contending with both DM and chronic lung disorders could markedly improve patient wellness and quality of life.

In summary, our findings align closely with existing systematic reviews and meta-analyses, reinforcing the association between DM and chronic lung diseases such as COPD and asthma. The evidence suggests that diabetes not only increases the risk of developing these conditions but also worsens their clinical course and lung function. Future studies should aim to clarify these associations and investigate potential interventions that address both diabetes management and respiratory health. While similarities exist, differences in focus and methodology highlight the need for further research to explore the complex relationships between diabetes and various chronic lung diseases like asthma and COPD comprehensively.

Strengths and limitations

One of the strengths of this review is its comprehensive approach, synthesizing findings from multiple studies to present a holistic view of the interrelationship between DM and chronic lung diseases. By employing a systematic search strategy across multiple databases, including PubMed, Cochrane Library, and Europe PMC, the review captures a wide range of patient outcomes and clinical data. This broad search enhances the robustness of the findings and provides a more complete understanding of the implications of diabetes beyond the commonly studied COPD and asthma.

However, there are limitations to consider. The reliance on observational studies may introduce biases, such as confounding factors, that can affect the reported associations. Additionally, the variation in study designs, sample sizes, and populations across the included articles may limit the generalizability of the findings. There is also a potential publication bias, as studies with significant results are more likely to be published. One notable limitation of this review is that it is not a systematic review. Consequently, we did not conduct a formal quality assessment of the included studies. This may affect the robustness of our findings and interpretations. Lastly, the review primarily focuses on studies published in English, which may exclude relevant research in other languages, potentially leading to gaps in the literature. Future reviews should consider incorporating non-English studies to provide a more comprehensive picture of this important health issue and consider employing systematic review methodologies to ensure a comprehensive evaluation of the quality of evidence.

Future research

Future research should aim to clarify the underlying mechanisms linking diabetes and chronic lung diseases, particularly through longitudinal studies that can establish causal relationships. Investigating the role of genetic and environmental factors in this relationship is also essential, as these factors may contribute to the shared risks of developing both conditions. Additionally, randomized controlled trials exploring integrated treatment strategies that address both diabetes management and respiratory health could provide valuable insights into improving patient outcomes. Finally, further studies should evaluate the impact of lifestyle interventions on reducing the risk of comorbidities in patients with diabetes and chronic lung diseases.

## Conclusions

In conclusion, this comprehensive review underscores the significant association between DM and chronic lung diseases, highlighting a bidirectional relationship that warrants attention in clinical practice. The evidence suggests that diabetes not only increases the risk of developing conditions such as COPD and asthma but also worsens their clinical course and lung function. Given the shared inflammatory pathways and the impact of comorbidities on patient outcomes, an integrated approach to managing both diabetes and chronic lung diseases is essential. Future research should focus on elucidating the mechanisms behind these associations and paving the path for developing targeted interventions that will improve the quality of life for affected individuals. The inclusion of the 10 studies analyzed provides a robust foundation for understanding these complex interrelationships.
